# Monitoring the durability of the long-lasting insecticidal nets Olyset^®^ and PermaNet^®^ 2.0 in similar use environments in Zanzibar

**DOI:** 10.1186/s12936-020-03258-w

**Published:** 2020-05-24

**Authors:** Khamis Ameir Haji, Bakari Omar Khatib, Emmanuel Obi, Kanuth Dimoso, Hannah Koenker, Stella Babalola, George Greer, Naomi Serbantez, Faiza Abbas, Abdullah Ali, Sean Blaufuss, Bolanle Olapeju, Albert Kilian

**Affiliations:** 1 Zanzibar Malaria Elimination Programme, Stone Town, Zanzibar, Tanzania; 2 PMI VectorWorks Project, Tropical Health LLP, Abuja, Nigeria; 3 PMI VectorWorks Project, JHU Center for Communication Programs, Dar es Salaam, Tanzania; 4 PMI VectorWorks Project, JHU Center for Communication Programs, Baltimore, MD USA; 5 U.S. President’s Malaria Initiative, U.S. Agency for International Development, Dar es Salaam, Tanzania; 6 PMI VectorWorks Project, Tropical Health LLP, Montagut, Spain

**Keywords:** LLIN, Durability, Monitoring, Zanzibar

## Abstract

**Background:**

Malaria transmission in Zanzibar has dramatically reduced in recent years but vector control interventions such as long-lasting insecticidal nets (LLIN) must continue to reach malaria elimination. To achieve this, the Zanzibar Malaria Elimination Programme needs actionable evidence of the durability of the LLIN brands distributed. This study compared physical and insecticidal durability of two LLIN brands: Olyset^®^ and PermaNet**©** 2.0 in two similar districts on the islands of Unguja and Pemba.

**Methods:**

This was a prospective cohort study of representative samples of households from two districts, recruited at baseline 4 months after the mass campaign. All campaign nets in these households were labelled and followed up over a period of 33 months. Primary outcome was the “proportion of nets surviving in serviceable condition” based on attrition and integrity measures and the median survival in years. The outcome for insecticidal durability was determined by bio-assay from sub-samples of campaign nets.

**Results:**

A total of 834 campaign nets (121% of target) from 299 households were included in the study. Definite outcomes could be determined for 86% of the cohort nets in Unguja (PermaNet^®^ 2.0) and 89% in Pemba (Olyset^®^). After 33 months, physical survival in serviceable condition was 55% in Unguja and 51% in Pemba. Estimated median survival was lower in Pemba at all time points with 2.3–2.7 years compared to 3.1–3.3 yeas in Unguja. Multivariable Cox proportionate hazard models confirmed the difference between brands (p < 0.0001) and identified household net-care attitude (p = 0.007) and folding of hanging nets during the day (p < 0.0001) as significant determinants, in addition to exclusive use of nets by adults (p = 0.03) and use only over a finished bedframe (p = 0.01). Optimal insecticidal effectiveness was 80% or higher for both brands at all time points when both cone bio-assays and tunnel tests were applied.

**Conclusions:**

After 3 years of follow-up, Olyset^®^ LLIN showed significantly lower physical survival compared to PermaNet^®^ 2.0 LLIN even after adjusting for other variables of net-use environment and net handling. This suggests that the differences were driven by the textile characteristics of the LLIN brands.

## Background

Zanzibar, the partly autonomous region of the United Republic of Tanzania, consists of the two islands of Unguja and Pemba. For many years malaria, mainly *Plasmodium falciparum*, has been endemic in Zanzibar. With the introduction of artemisinin-based combination therapy in 2003 and mass distribution of long-lasting insecticidal nets (LLIN) in 2006, malaria case incidence and transmission have dramatically reduced [1, 2]. However, the prospect of malaria elimination is threatened by emerging resistance of the vectors [3] and importation of malaria from mainland Tanzania [4]. Therefore, comprehensive vector control will have to continue. To maintain universal coverage with LLIN it is crucial to understand the physical and insecticidal durability of the LLIN brands distributed in order to determine when and how many nets will need to be replaced. In 2014, the World Health Organization (WHO) issued a comprehensive guidance on how best to undertake physical and insecticidal durability monitoring of LLINs in a standardized fashion [5, 6]. The first large-scale LLIN durability study in Tanzania using this new methodology was undertaken 2013–2016 in eight districts of mainland Tanzania and had two main components. First, a retrospective durability assessment of Olyset^®^ distributed through mass campaigns between 2009 and 2011, and second, a 3-year prospective study of three LLIN brands, Olyset^®^, Netprotect^®^ and PermaNet^®^ 2.0 [7]. Findings from the retrospective study suggested that median survival of Olyset^®^ in mainland Tanzania might be below the assumed 3-year level [8]. This was then confirmed in the prospective study where median survival estimates of 2.0 years were calculated for Olyset^®^, 2.5 years for PermaNet^®^ 2.0 and 2.6 years for Netprotect^®^ [9].

LLIN durability data for Zanzibar are scarce. A first assessment was carried out in 2011 looking at Olyset^®^ LLIN distributed in 2008. The study did not yet use the WHO recommended methodology and found that 68% of the nets were ‘damaged’ after 3 years [3]. In June 2015, the vector control unit of the Zanzibar Malaria Elimination Programme (ZAMEP) undertook a cross-sectional, retrospective, durability assessment of Olyset^®^ LLIN that had been distributed 3 years earlier, in 2012 (ZAMEP, unpublished). This study covered all 10 districts of Zanzibar. Out of 250 nets sampled, 74% were still in use, 20% had been lost due to damage and 6% were still in their original package. Some 90% of the nets had any holes, but the study did not calculate the proportionate hole index so that the proportion of nets in serviceable condition is not reported. Bio-assays of the Olyset^®^ samples using the cone and tunnel tests showed that while mortality of pyrethroid sensitive *Anopheles gambiae* sensu stricto (s.s.) was only 50% in cone assays, it was 80% in the tunnel test.

In 2016 ZAMEP and its partners launched a repeat mass campaign to maintain universal coverage with LLIN where multiple brands of LLIN were distributed. This was in addition to ongoing distribution through antenatal care and immunization services, as well as through a community distribution channel. In line with the need of ZAMEP to obtain actionable evidence of the durability of LLIN in Zanzibar, the objectives of the present study were to (i) compare physical and insecticidal durability of two LLIN brands, Olyset^®^ and PermaNet**©** 2.0, distributed during the 2016 mass campaign, in two similar districts on the islands of Unguja and Pemba using a prospective cohort study design; and, (ii) identify major determinants influencing LLIN durability.

## Methods

### Study sites

**Fig. 1:**
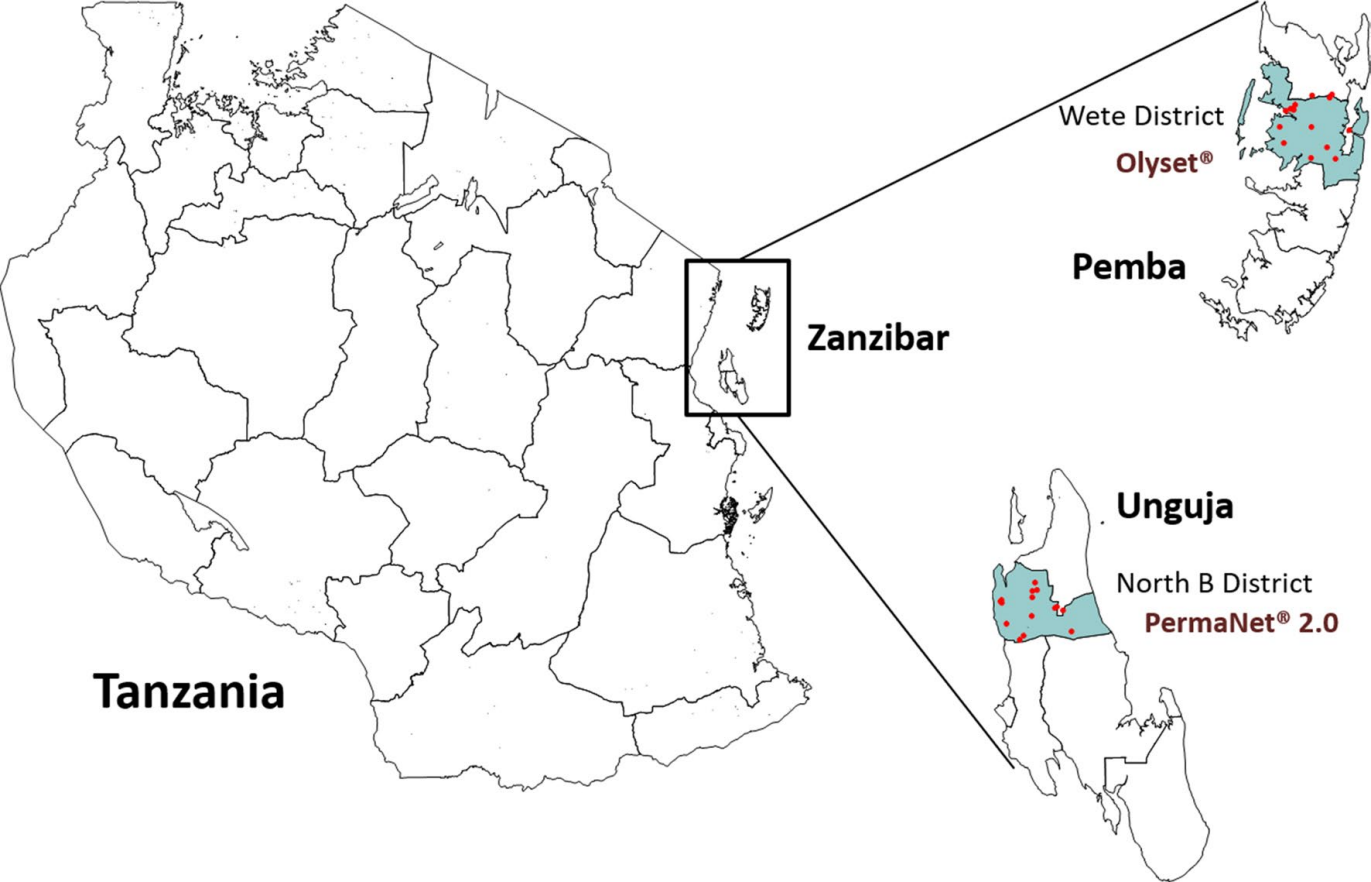
Location of study sites within Zanzibar with sampled clusters (dots)

Two districts with a similar environment were selected: Wete District on Pemba and North B District on Unguja. They are shown in Fig. 1 with the geo-location of the study clusters. Both districts are located in the northern region of the respective islands (Kaskazini Pemba and Kaskazini Unguja) and have an estimated 2016 population of 121,000 (Wete) and 50,000 (North B). The climate is equatorial (warm and humid), with a bi-modal rain pattern: the first rainy season lasts from March to May and the second rainy season from October to December with an average annual rain fall of 1500–1700 mm. Malaria parasite prevalence was estimated by microscopy as 0.5% in North B district and 1.1% in Wete in the 2015–2016 Demographic and Health Survey [10].

### Study design

This was a prospective study of representative cohorts of LLINs distributed during the 2016 mass distribution campaign and followed for up to 3 years. The design was based on the guidance from the US President’s Malaria Initiative for LLIN durability monitoring [11] and in this case comparing the durability of the two different LLIN brands between the two very similar locations. The first brand was PermaNet^®^ 2.0, a 100-denier polyester LLIN in blue and white colour and distributed in Pemba. This LLIN uses the coating technology with a loading dose of 55 mg/sq m of deltamethrin and obtained interim WHOPES recommendation in December 2008 [12] and WHO prequalification in December 2017 [13]. The second LLIN brand was Olyset©, a 150-denier polyethylene LLIN in blue colour which uses incorporation technology with a loading dose of 1000 mg/sq m of permethrin. Olyset© received full WHOPES recommendation in July 2013 [14] and WHO pre-qualification status in December 2017 [13].

Within 6 months of the respective mass distribution campaigns LLINs were to be sampled and followed up after 12, 24, and 36 months through household surveys. At each time point measures of physical durability were assessed (attrition and integrity) using a household questionnaire and net damage assessment tools. For all data points after baseline, 30 campaign nets per site were sampled and retrieved for assessment of insecticidal effectiveness (bio-assay) as recommended by the WHO [15].

### Sample size and sampling

Sample size was targeted at finding a difference of ± 9%-points from a 50% LLIN survival point estimate after 3 years as significant at the 95% confidence level or an 18% difference between the two LLIN brands. This is equivalent to a deviation from the assumed 3-year median survival by 10–12 months. Further assumptions were a power of 80%, design effect of 2.5, all-cause attrition of 35% and attrition due to wear and tear of 20% over 3 years [16], an initial household non-response rate of 5%, campaign distribution of one LLIN for every two people with rounding up for odd-numbered households, and an initial loss between campaign and baseline survey of 8% of the campaign nets. This resulted in the need for a cohort of 345 campaign nets to be recruited per site. Based on an estimated average household size of five persons this required 150 households sampled from 15 clusters with 10 households each.

First, clusters were sampled with probability proportionate to size using the campaign registration lists as sampling frame. Since these lists were only available for *shehias* (administrative unit equivalent to a ward), these were selected and then one community within the *shehia* was sampled using simple random selection. Second, households within clusters were selected using simple random sampling from lists of eligible households prepared by the field teams on the day of the survey. For communities with more than 200 households a segmentation approach was used and only the randomly selected segment was sampled. Up to five replacement households were sampled per cluster to substitute in case a household had not received nets from the campaign or did not consent to participate. Within each household all LLINs identified as from the campaign by brand, colour and report by the respondent were labelled with a unique ID number and barcode for follow-up, even when they were still in the package at the time of the baseline survey.

Campaign nets for bio-assay testing were sampled from the cohort only at the final survey using simple random sampling. For the 12- and 24-months surveys campaign nets were sampled from neighbouring households as follows: within each cluster two or three index households were randomly identified from the cohort and when the field teams reached these study households, they went left to the nearest neighbour that had campaign nets and consented to give them up for the study. A brief questionnaire was filled for these nets regarding use and washing. For all LLINs collected for bio-assay new replacement LLINs were given.

### Field procedures

An implementation team of nine individuals was established per site, with one overall site coordinator and two field teams each consisting of one supervisor and three interviewers. Activities in the field were overseen by staff from ZAMEP. Interviewers and supervisors were carefully selected so that they were culturally acceptable, had good knowledge of the local languages and experience in conducting household surveys. A 5-day training was held at baseline and 3-day refresher training was done before each follow-up survey. Special emphasis was put on the process of a standardized assessment of net damage using a template to identify hole size categories and tallying hole counts using an application on the digital devices used for data entry. The questionnaire had three main modules: one for the household respondent, a second for the cohort campaign nets (including nets lost between campaign and baseline survey), and a third module for other nets owned by the household at each time point. In addition, a list of household members and assets was obtained at baseline and at the final survey. GPS coordinates were recorded at baseline and used to track households during follow up. If households moved within the clusters the new homes were identified, if they moved outside the cluster, they were considered lost to follow-up. The questionnaire and all other tools are publicly available (http://www.durabilitymonitoring.org).

The mass distribution campaign took place mid July 2016 at both sites. Baseline assessment was done in November 2016, the 12 months data collection was carried out July/August 2017, the 24 months surveys were done in June/July 2018, and the final survey took place April 2019. The earlier date for the last survey was chosen in view of the pending close-out of the PMI VectorWorks project.

### Laboratory analysis

Bio-assays were done at the ZAMEP facilities in Unguja (cone tests) and Pemba (tunnel test) using the standard WHO bio-assay test procedures [15]. Five non-blood-fed, 2–5-day-old females of the known susceptible *An. gambiae* s.s. R.70 strain maintained at ZAMEP insectary were exposed for 3 min in each cone and then held for 24 h with access to sugar solution. Five sites were tested on each net (4 sides and roof panels) and two replicates per location (10 cone tests with 50 mosquitoes per net in total). Knockdown was measured 60 min after exposure and mortality was scored after 24 h. A negative control, from an untreated net, was included in each round of cone bio-assay testing. Bio-assays were carried out at 27 ± 2 °C and 80 ± 10% relative humidity. Recorded were 60-min knock-down (KD60) and 24-h mortality and then combined as optimal insecticidal effectiveness (KD60 ≥ 95% or mortality ≥ 80%), minimal effectiveness (KD60 ≥ 75% or mortality ≥ 50%), or failure (not reaching minimal effectiveness criteria) [5].

For the tunnel test the netting piece that resulted in mortality close to the average mortality in the cone bio-assay was selected. In each netting sample, 9 holes were cut measuring 1 cm in diameter, one hole was located at the centre of the square, and the other eight were at the same distant and located 5 cm from the border. The LLIN piece was then held in a disposable cardboard frame.

In the shorter section of the tunnel, a rabbit was tightly held and unable to move at 18 h up to the end of experiment on the following day at 09.00 h. One-hundred female, non-blood-fed, susceptible *An. gambiae* s.s. aged between 5 and 8 days were introduced into the cage at the end of the longer section of the tunnel. Mosquitoes were free to fly in the tunnel but had to make contact with the piece of netting and locate the holes in it before passing through to reach the bait in a shorter section of the tunnel. A tunnel with untreated netting piece with holes was used as a negative control during the test. The tunnels tests were carried out at 27 ± 2 °C and 75% ± 10% relative humidity at night in full darkness. At the end of experiment, the mosquitoes were removed from each section of the tunnel using sucking tube and counted separately; mortality and blood-feeding rates were recorded. Blood-feeding inhibition was assessed by comparing the proportion of blood-fed females (alive or dead) in treated and control tunnels. Overall mortality was measured by pooling the mortality rates of mosquitoes from the two sections of the tunnel. For the evaluation of the tunnel test the following criteria were used: optimal effectiveness: ≥ 80% mortality or ≥ 90% blood-feeding inhibition.

### Data management

For data collection, tablet PCs were used and installed with the Open Data Kit (ODK) software for the questionnaire and Open Street Map for Android (OSMAND) for household tracking. Data from each field team was collected daily and directly uploaded to a secure data base if internet was available or collected on a local storage device by the coordinator until it could be transferred. Data were converted from ODK to comma-delimited data files using the ODK briefcase tool for inspection of incoming data and daily feedback on data quality was provided to the teams. For each survey round, updated lists were compiled from the household and cohort net master files and preloaded on the ODK system including all households and cohort nets for which no definite outcome was available to date. After completion of the surveys, datasets were transferred to Stata version 14.2 (Stata, Texas, USA) for further aggregation, consistency checks and preparation for analysis. Stata do-files (macros) developed by the PMI VectorWorks project were applied and adjusted as needed [11]. For the final analysis data sets from all four surveys were merged.

### Data analysis

#### Definition of outcomes

The primary outcome measure was the *physical net survival* and was defined as the proportion of cohort nets received from the LLIN campaign still in serviceable physical condition (definition provided below) [5]. For the calculation of this outcome two interim outcomes were calculated as follows:

*Net attrition rate due to wear and tear* was defined as the proportion of originally received nets which were lost due to wear and tear (thrown away, destroyed or used for other purposes) at the time of assessment. Nets received but given away for use by others or stolen were excluded from the denominator. Similarly, nets with unknown outcome were excluded.

*Net integrity* was measured first by the proportionate hole index (pHI) as recommended by WHO [15]. Holes in cohort LLINs were counted and categorized into four different sizes: size 1, 0.5–2 cm, size 2: 2–10 cm, size 3: 10–25 cm and size 4: larger than 25 cm in diameter. The proportionate pHI for each net was then calculated as the number of holes counted multiplied by the size category weights as suggested by WHO [15]. Based on the pHI each net was then categorized as ‘good’, ‘damaged’, ‘serviceable’ or ‘torn’ as follows [15]:

Good: total hole surface area < 0.01 sq m or pHI < 64
Damaged: total hole surface area 0.01–0.1 sq m or pHI 65–642
Torn: total hole surface area > 0.1 sq m or pHI > 642
Serviceable: total hole surface area ≤ 0.1 sq m or pHI ≤ 642 (good or damaged)

In order to be able to compare physical survival measured at different time points the outcome of *median net survival* was estimated defined as the time in years until 50% of the originally distributed LLINs were no longer serviceable. Two approaches were used to estimate median survival. At each time point the proportion surviving in serviceable condition was plotted against time of follow up and compared to the hypothetical survival curves with defined median survival [1]. The median survival estimate was taken as the interpolated position of the data point on a horizontal line between the two adjacent median survival curves. After the final survey median net survival was calculated from at the last two time points provided both were below 85% (when the hypothetical curves are linear), using the following formula:

$${\text{tm}} = {\text{t}}1 + \frac{{\left( {{\text{t}}2 - {\text{t}}1} \right) *\left( {{\text{p}}1 - 50} \right)}}{{\left( {{\text{p}}1 - {\text{p}}2} \right)}}$$

where tm is the median survival time, t1 and t2 the first and second time points in years and p1 and p2 the proportion surviving to first and second time point respectively in per cent. Confidence intervals for this estimate were calculated by projecting the 95% CI from the survival estimates in the same way as described above.

### Explanatory variable preparation

Overall household attitudes towards net care and repair were measured using a set of Likert score questions where a statement was read to the respondent (head of household or spouse) and the level of agreement recorded. These were analysed by recoding the four-level Likert scale score to have a value of − 2 for ‘strongly disagree’, − 1 for ‘disagree’, + 1 for ‘agree’ and + 2 for ‘strongly agree’. These attitude scores for each respondent were then summed and divided by the number of statements to calculate an average household attitude score for which 0 represents a neutral result and positive values a positive result. For each site the proportion of households with a score above 1 (very positive attitude) were calculated at each survey.

Further aggregation of results was done across all four surveys. For household and net risk factors for durability the following categories were used: ‘never’ = responded with ‘never’ in all surveys the household participated; ‘at times’ = household reported the behaviour as ‘sometimes’ in at least one survey round or had conflicting statements; ‘always’ = responded with ‘always’ in all surveys the household participated. Exposure and attitude were similarly aggregated, i.e., ‘once’ = reported exposure or positive attitude score at one of the four survey rounds; ‘twice or more’ = at two or more survey rounds.

A wealth index was calculated for the baseline data set using the basic household assets and a principal component analysis with the first component used as the index. Households were then grouped into tertiles. The full household data collection and wealth index was repeated at the final survey. However, at the 12 and 24 months no specific household or member data were collected.

### Statistical analysis

For continuous variables, arithmetic means were used to describe the central tendency and the t-test for comparison of groups for normally distributed data. Otherwise, median and Kruskal–Wallis test were used. Proportions were compared by contingency tables and the Chi-squared test was used to test for differences in proportions. For calculation of confidence intervals around estimates, the intra- and between-cluster correlation has been taken into account. Data were set up for survival analysis as a duration format dataset where each time interval for a net is a separate observation. Analysis was done using an intention to treat approach, i.e., risk of failure was considered to start at the day of distribution irrespective of whether or when the net was hung and used. Failure was defined as a net being lost to wear and tear or torn based on physical assessment (pHI). The time of failure was directly calculated from the report of time of loss by the respondent or taken as the mid-point between the last two surveys if unknown. A secondary analysis used a per-protocol approach where the risk of damage was considered to begin only when a net was first hung. Basic survival analysis was done using Kaplan–Meier estimations of survival function. Determinants of survival were explored using Cox proportionate hazard models. Separate models were constructed for household factors and for net level factors, such that models with net-level factors included only nets that had been ever hung for use during the study. Factors were tested first in individual models which were then used to construct the final multivariable models. Final model fit was tested using a linktest and Schoenfeld residuals and log–log plots were used to check the proportionate hazard assumption.

## Results

### Sample characteristics

A cohort of 834 campaign nets from 299 households was recruited at baseline representing 121% of targeted sample size for cohort nets. This was in part due to a slightly higher than expected average household size of 5.4 persons in Unguja and 6.1 in Pemba, but also to slightly more nets than expected delivered by the campaign with on average one campaign net per 1.83 household members in Unguja and 1.92 in Pemba. Details of the follow-up status of households and cohort nets are given in Table 1. For 86% of the cohort nets in Unguja a definite outcome could be determined and for 89% in Pemba. The most common reason of an unknown outcome was that the net was not present at the last survey but the respondent did not know what had happened to it. Household mobility was low at both sites with only 5% of households moving away in Unguja and 2% in Pemba. Only four households refused further participation, three in Unguja and one in Pemba.

**Table 1:** Follow-up status of recruited households and campaign cohort nets after final survey

Variable	Unguja	Pemba
Households	N = 149	N = 150
Still has any campaign nets	71.1%	74.7%
Lost all their campaign nets	20.8%	16.7%
Moved away	4.7%	2.2%
Refused	2.0%	0.7%
Nobody home at survey	1.3%	2.0%

Campaign cohort nets	N = 382	N = 452
Known outcome	85.9%	89.4%
Unknown outcome	14.1%	10.6%
Household moved away or refused	5.8%	4.7%
Net used elsewhere	1.1%	0.7%
Fate of net unknown	7.3%	5.3%

There was no evidence that the demographic and socio-economic characteristics of households in either of the sites had changed significantly during the study period and overall both sites were very similar (details shown in Additional file 1). The proportion of female-headed households was slightly higher in Unguja than Pemba (24 vs 11%, p = 0.016) and household size marginally higher in Pemba than in Unguja (6.1 vs 5.4, p = 0.053). The education status of heads of households was similar between sites with 27% non-literate, 30% primary and 43% secondary or higher education, but educational status was lower for female compared to male heads of households (p < 0.0001) with 47% of female heads non-literate in Unguja and 65% in Pemba.

Access to safe drinking water and latrines was over 80%, including 39% of households that had improved pit latrines or flush toilets. House characteristics were also similar for both sites. The vast majority of roofs were grass or thatch (98%), walls mostly plastered or brick (72%), and floors made from tiles (72%). Fuel for cooking was predominantly firewood in Unguja (93%), but in Pemba only 78% used firewood and 21% used charcoal (p = 0.02). Less than 1% of households in either site used kerosene or gas for cooking. Households owned a considerable variety of assets with the most common being mobile phones (89% in Unguja and 85% in Pemba). Ownership of radios was higher in Unguja compared to Pemba (65 vs 44%, p = 0.0001) but television sets were more common in Pemba (19 vs 36%, respectively, p = 0.04). About one in every six households in Unguja and one in every four in Pemba owned items such as a fridge, fan, iron, or smartphone. Means of transport available to households were the same in both sites with 59% owning bicycles, 12% motorbikes and 4% cars. Only one household in Pemba also owned a boat. The economic situation was that of primarily subsistence farming communities. Only 19% of households in Unguja and 14% in Pemba did not have either land to farm or some livestock, while about half (48% in Unguja and 46% in Pemba) had both. Livestock ownership was very similar between sites comprising mainly of chicken (60%), cows (12%), ducks or turkeys (9%), and goats (5%).

### Risk factors of physical durability

Household-related factors that are known or suspected to be causally linked to physical durability depended exclusively on the recall of the survey respondents. These were in 27% the head of household, in 58% the spouse and in 15% another adult family member. Seeing rodents or their traces was ubiquitous but varied slightly between surveys with on average 90% in Unguja reporting their presence and 82% in Unguja. Other key risk factor variables are presented in Table 2. The vast majority of households stored food in their sleeping rooms at least sometimes and close to half reported doing so all the time. This is thought to attract rodents to the rooms where the nets are and increase the risk of damage. Cooking in the sleeping rooms differed between the sites, 86% of households in Pemba never reported doing so while this rate was only 58% in Unguja where 32% of households cooked in sleeping rooms at times compared to only 7% in Pemba.

**Table 2:** Net-use environment at household

Variable	Unguja	Pemba	p-value for site comparison
Households	N = 149 % (95% CI)	N = 150 % (95% CI)	
Storing of food in sleeping rooms			
Never	4.7 (2.2–9.6)	3.3 (1.2–8.7)	0.64
At times	53.2 (45.9–60.0)	49.3 (39.0–59.7)	
Always	42.3 (36.5–48.3)	47.3 (36.6–58.3)	
Cooking in sleeping room			
Never	57.7 (51.0–64.2)	86.0 (74.2–92.9)	0.003
At times	31.5 (27.0–36.5)	7.3 (4.1–12.8)	
Always	10.7 (6.3–17.6)	6.7 (2.3–17.6)	
Exposure to net use or care messages			
Never	67.1 (58.0–75.1)	44.0 (31.9–56.9)	0.002
Once	24.8 (17.9–33.3)	34.0 (27.3–41.5)	
Twice or more	8.0 (4.7–13.3)	22.0 (15.2–30.8)	
Very positive net care attitude (score > 1.0)			
Never	35.6 (26.5–45.8)	46.0 (33.3–59.2)	0.046
Once	36.9 (28.8–45.8)	42.0 (31.3–53.6)	
Twice or more	27.5 (19.8–36.8)	12.0 (6.1–22.2)	

Results were aggregated across all four surveys i.e., ‘never’ = household did not report the behaviour at any survey round; ‘at times’ = household reported the behaviour as ‘sometimes’ in at least one survey round or gave conflicting information; ‘always’ = reporting ‘always’ at all surveys. Exposure and attitude were similarly aggregated, i.e., ‘once’ = reported exposure or positive attitude score at one of the four survey rounds; ‘twice or more’ = at two or more survey rounds

Exposure to net-related messages was generally low, fluctuating between 11 and 33% at any time point, but better in Pemba with 56% of household respondents in Pemba reporting any exposure at any of the surveys compared to only 33% of households in Unguja (p = 0.005). Social and behaviour change (SBC) communication was almost exclusively through inter-personal communication (IPC), mainly through facility and community health workers and to some extent through community leaders (28%), with the exception of baseline when 43% of households reported being exposed to messages through radio. In contrast to message exposure, net-care attitude scores were slightly higher in Unguja than in Pemba with 64% of households having been scored as ‘very positive attitude’ at least once during the study compared to 54% in Pemba and 27 and 12%, respectively, at least twice.

Durability risk factors regarding the handling and use of nets when hanging are shown in Table 3. Out of the eight criteria considered, two: type of sleeping place and ever washing of nets, were the same in both sites. Another three were statistically moderately different with slightly higher proportion of cohort nets used by adults in Unguja, slightly more nets always washed with detergents in Pemba and slightly more nets at least sometimes dried over bushes or fences in Unguja. However, these differences were programmatically less relevant as the vast majority of cohort nets were washed with detergent and never dried over bushes. Stronger evidence for differences existed for average washing frequency in the last 6 months which was higher in Unguja by 0.6 washes per year, and for always drying washed nets outside which was 13% points higher in Unguja. The biggest difference with respect to potential impact on physical durability was seen for folding or tying nets up during the day when they were hanging and here particularly the proportion of nets always found folded or tied which was 29% in Unguja and 51% in Pemba. This difference was driven by a significant increase of hanging nets found folded or tied in the last two surveys in Pemba which had increased from 54% at baseline and 59% at 12 months to 91% at the 24 and 36 months survey. In contrast the rate of folding at each survey remained between 49 and 68% in Unguja.

**Table 3:** Net-use environment and washing of cohort nets from campaign

Variable	Unguja	Pemba	p-value for site comparison
Cohort nets	N = 382 % (95% CI)	N = 452 % (95% CI)	
Ever found hanging	77.8 (69.8–84.1)	76.3 (67.7–83.2)	0.82
Ever used	77.5 (69.8–83.7)	75.9 (45.9–63.6)	0.73

Cohort nets ever hung	N = 325	N = 372	
Tied up or folded when hanging			
Never	32.9 (21.6–46.7)	20.7 (15.6–26.9)	0.010
At times	38.2 (30.0–47.0)	28.0 (19.1–38.9)	
Always	28.9 (20.3–39.5)	51.3 (38.9–63.7)	
Type of sleeping place^a^			
Bed frame (finished)	26.3 (16.5–39.2)	27.8 (16.8–42.4)	0.55
Bed frame (sticks)	54.6 (42.2–66.5)	51.3 (39.5–63.0)	
Foam mattress	15.4 (10.9–21.3)	19.4 (15.7–23.7)	
Reed mat	3.8 (1.3–10.1)	1.5 (0.5–3.9)	

Cohort nets ever used	N = 273	N = 321	
Dominant user group			
Children only	16.1 (11.6–22.0)	24.9 (19.1–31.8)	0.075
Children with adults	26.7 (20.7–33.8)	26.5 (21.6–32.0)	
Adults only	57.1 (51.5–62.6)	48.6 (40.3–57.0)	
Ever washed	93.6 (85.6–97.3)	94.5 (90.2–96.9)	0.77

Cohort nets ever washed	N = 296	N = 338	
Washes last 6 months^b^			
Median (IQR)	2.3 (1.7–3.0)	2.0 (1.5–3.0)	0.001
Use of detergent			
Never	0 (–)	1.2 (0.4–3.8)	0.080
At times	10.8 (6.3–18.0)	4.1 (1.4–11.5)	
Always	89.2 (82.0–93.7)	94.7 (87.0–98.0)	
Drying net outside			
Never	0 (–)	4.4 (2.3–8.5)	0.009
At times	5.7 (2.7–11.8)	14.2 (8.4–23.0)	
Always	94.3 (88.2–97.3)	81.4 (69.6–89.3)	
Drying over bush or fence			
Never	93.2 (86.3–97.0)	98.5 (94.9–99.6)	0.033
At times	6.4 (3.1–12.7)	1.5 (0.4–5.1)	
Always	0.3 (0.0–2.5)	0 (–)	

^a^Lowest type of sleeping place ever reported for net

^b^Average of all recoded 6 months episodes for each net

**Fig. 2:**
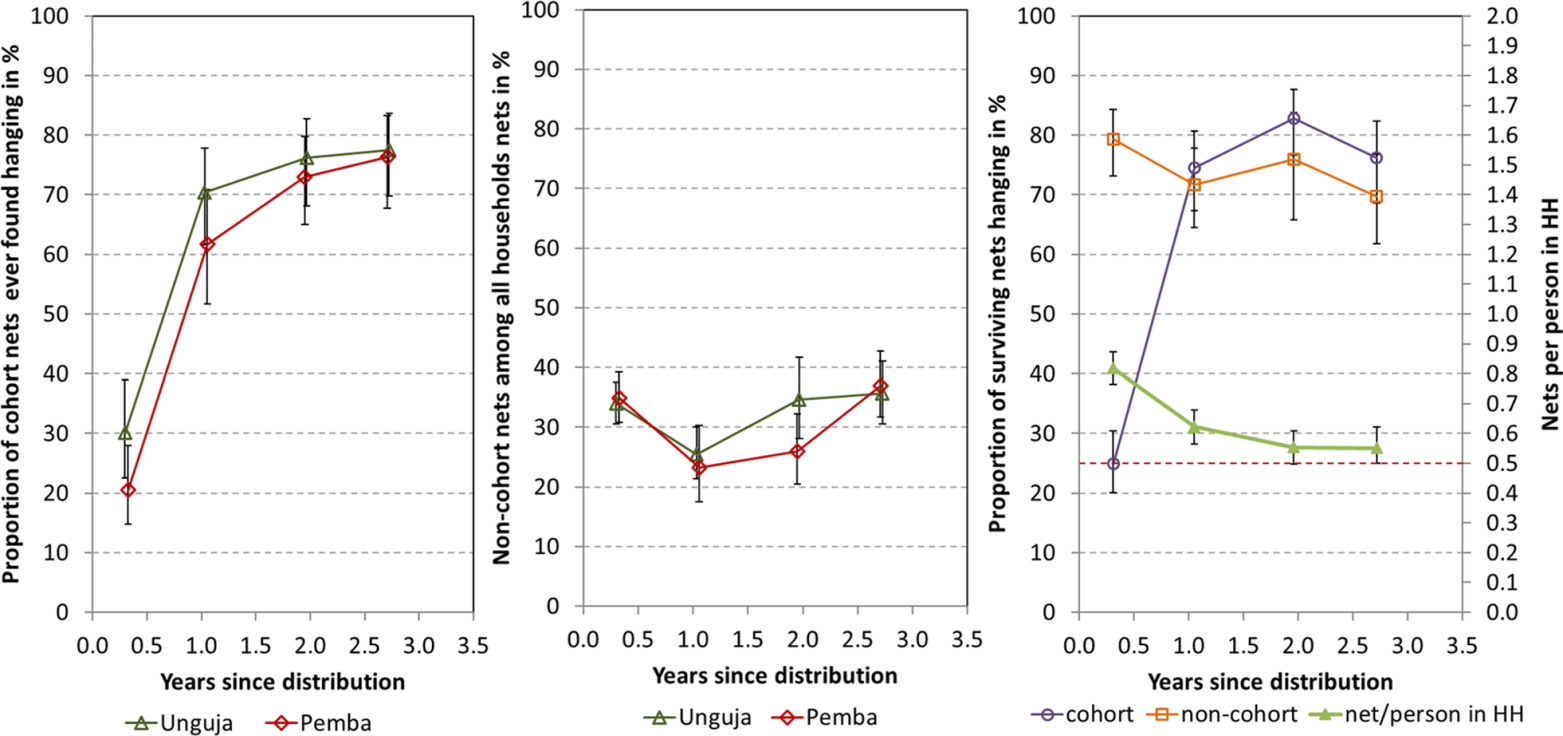
Hanging of cohort and non-cohort nets. Left: proportion of sampled cohort nets ever found hanging; middle: proportion of non-cohort nets among all nets owned by households; left: proportion of all surviving nets hanging against number of nets owned by household; dashed red line indicates level of 1 net/2 people

At baseline, around 4 months after the campaign, only 30% of the cohort nets in Unguja and 21% in Pemba were found hanging while 67 and 77%, respectively, were still in their packages. The proportion of cohort nets ever found hanging thereafter increased rapidly to reach 76% after 24 months in Unguja and 73% in Pemba (Fig. 2, left). There was minimal further increase in ever hanging at 36 months (Table 3) and overall there was no difference in cohort nets ever found hanging between the sites. At baseline, 34% of households in Unguja and 35% in Pemba also owned other nets not obtained from the recent mass campaign and of these nets 84% in Unguja and 66% in Pemba were hanging. Over the follow-up period households owning any non-cohort nets dropped after baseline to 21% in Unguja and 18% in Pemba but then steadily increased again with influx of new nets reaching 36% at both sites at the final survey (Fig. 2, middle). These new nets came predominantly from the public sector (82%) with 9% from the markets and 8% from family or friends. The dynamic between the overall household ownership of any nets, measured as mean nets per person in the household, and hanging of cohort and non-cohort nets over time is shown in Fig. 2(right) and suggests that initially cohort nets were not used because these was a certain oversupply following the campaign and households first used the nets they still had before switching to the new nets.

Use of cohort as well as non-cohort nets was strongly associated with nets hanging, i.e., 96% of nets that were observed hanging during the surveys were reported to have been used the previous night while only 5% of those not hanging had been used. There was some evidence of seasonal variation in net use in Pemba where 73% of household respondents said they used the nets equally during the rains and the dry season, 25% mainly in the rainy season and 2% only during the rains. In Unguja the respective proportions were 86, 8 and 6% (p = 0.0004 for site comparison).

### Attrition

**Fig. 3:**
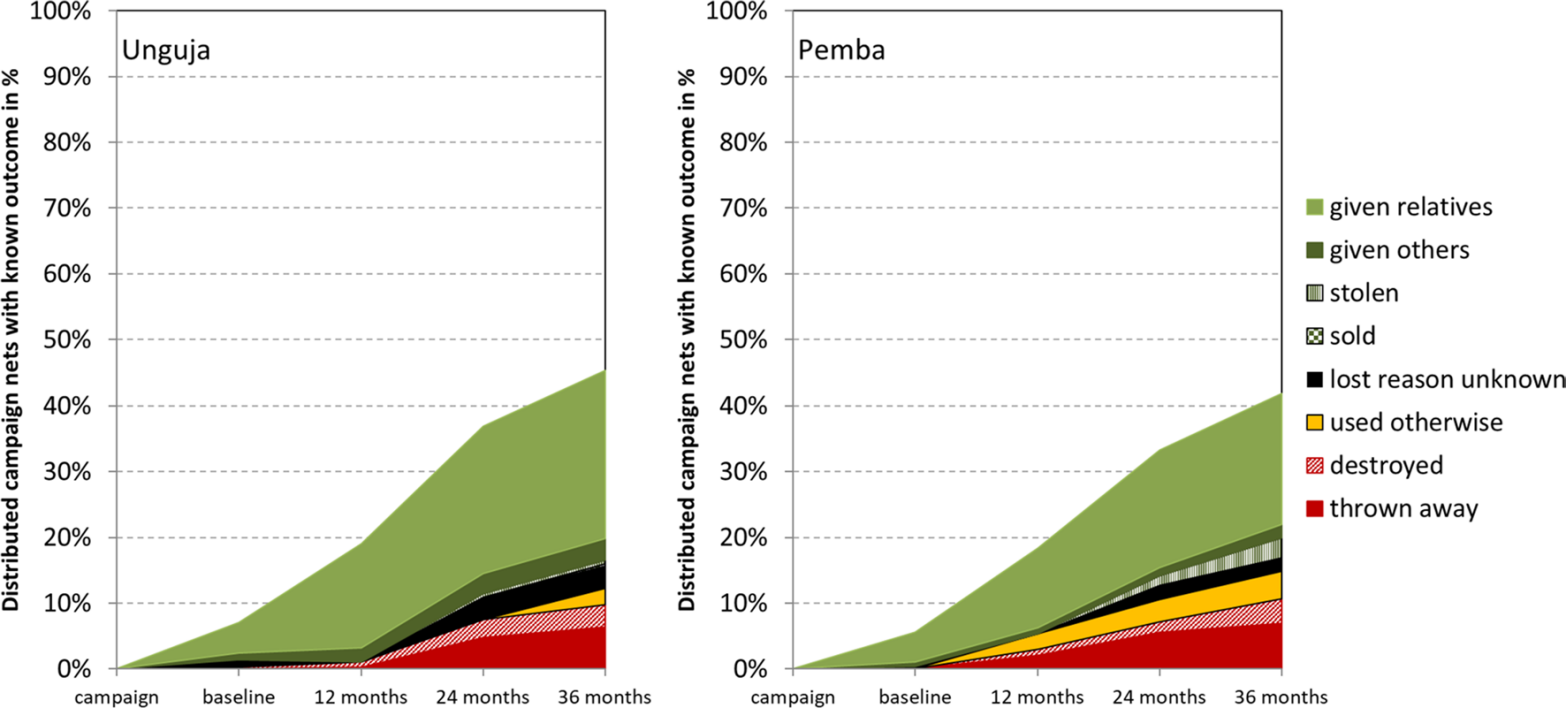
Attrition of cohort nets and their causes

All-cause attrition of cohort nets, i.e., losses for any reason, was similar at both sites increasing from 6% at baseline to 19% at 12 months, 35% at 24 months, and 44% at the final survey 33 months after distribution (p > 0.5 for site comparison at all time points). More importantly, losses due to wear and tear (destroying, throwing away or use for other purposes) were higher in Pemba compared to Unguja at the 12 months surveys with 5 vs 1.0% (p = 0.003) and remained higher at 24 and 33 months but the difference grew smaller with 11 vs 8% (p = 0.38) and 15 vs 12% (p = 0.52). Details of the reasons for loss are shown in Fig. 3. The proportion of losses due to wear and tear among all losses was very small at baseline (4% in Unguja and 0% in Pemba) meaning that almost all initial losses were due to nets being given away to relatives or others. The proportion gradually increased and at the final survey 27% of all-cause attrition was due to wear and tear in Unguja and 36% in Pemba. Two cohort nets in Unguja (0.6%) and 13 in Pemba (3%) were reported stolen and one cohort net in Unguja was sold (0.3%). Reasons for loss among those nets lost to wear and tear were similar between the sites (p = 0.81) with 49% destroyed, 26% thrown away, and 25% used for other purposes. Considering all cohort nets with known outcomes the proportion used for other purposes was 3%. The vast majority of these nets was used to protect crop (48%) or cut up for various other uses (41%). One net in Pemba was reportedly used for fishing, 4% of nets used for other purposes and 0.1% of all nets with known outcome.

### Integrity

Details of the physical assessment of cohort nets seen at each time point are presented in Table 4. As one would expect, the proportion of LLINs still present in the surveyed households with any sign of damage initially increased rapidly but then the increase slowed down as older nets were increasingly discarded. At the final survey 90% of nets in Unguja and 89% in Pemba had any holes and the level of damage was very similar in both sites based on median hole index of nets with any holes and the proportion of nets in good and serviceable condition (p = 0.8). The proportion of cohort nets in good and serviceable condition decreased over time while those damaged and torn increased without a significant difference between sites. The general damage pattern reported by households for each of the cohort nets with any damage was dominated by mechanical damage and was similar within each site varying between 57 and 78% at the different surveys, but differed between the sites. In Unguja there was a high level of rodent damage reported (48% at final survey) which was absent or minimal in Pemba (7 to 11%).

**Table 4:** Integrity of campaign nets present in households

Variable	Baseline % (95% CI)	12 months % (95% CI)	24 months % (95% CI)	36 months % (95% CI)
Unguja	N = 382	N = 305	N = 225	N = 195
Mean months since campaign	3.6	12.4	23.6	32.7
Net has any hole	9.9 (6.5–15.0)	48.5 (40.1–57.1)	78.2 (67.7–86.0)	89.7 (78.4–95.5)
Physical condition (pHI)				
Good (0–64)	96.3 (91.8–98.4)	78.4 (69.4–85.3)	50.2 (39.3–61.1)	33.3 (22.6–46.1)
Damaged (65–642)	2.9 (1.2–6.5)	16.7 (11.0–24.5)	34.7 (28.0–42.1)	34.4 (25.3–44.7)
Torn (> 642)	0.8 (0.2–3.6)	4.9 (2.5–9.5)	15.1 (9.7–22.9)	32.3 (22.7–43.7)
Serviceable (0–642)	99.2 (96.4–99.8)	95.1 (90.5–97.5)	84.9 (77.2–90.3)	67.7 (56.3–77.3)
Median pHI if any hole (IQR)	25 (2–200)	48 (23–233)	191 (30–520)	269 (59–1032)
Has any repairs if any hole	n/a	18.9 (11.3–30.0)	37.5 (29.0–46.9)	46.3 (35.8–57.1)

Pemba	N = 452	N = 352	N = 277	N = 250
Mean months since campaign	3.9	12.7	23.4	32.5
Net has any hole	7.3 (5.0–10.6)	54.8 (44.4–64.8)	75.8 (65.5–83.8)	88.8 (82.6–93.0)
Physical condition (pHI)				
Good (0–64)	96.5 (94.0–97.9)	74.7 (65.5–82.2)	49.1 (38.6–59.6)	34.8 (26.3–44.4)
Damaged (65–642)	2.7 (1.5–4.7)	17.1 (12.9–22.2)	28.5 (22.9–34.9)	29.2 (23.0–36.2)
Torn (> 642)	0.9 (0.3–2.3)	8.2 (4.6–14.4)	22.4 (16.7–29.4)	36.0 (29.5–43.0)
Serviceable (0–642)	99.1 (97.7–99.7)	91.8 (85.6–95.4)	77.6 (70.7–83.3)	64.0 (57.0–70.5)
Median pHI if any hole (IQR)	55 (4–248)	50 (6–343)	181 (29–772)	324 (57–1241)
Has any repairs if any hole	n/a	12.4 (6.0–24.0)	31.0 (19.8–44.9)	41.0 (30.6–52.3)

Observed partial or full repairs of holes increased with increasing damage at both sites and at the final survey 46% of cohort nets with any holes in Unguja and 41% in Pemba showed any sign of repair. The predominant method of repairing holes was stitching in Pemba with 91% of reported households that had done any repairs compared to 12% by knotting (some households used both methods of repair) while in Unguja it was 59 and 57%, respectively. No patching was used in either site and repairs were exclusively done by family members or by relatives or friends. Households with hole experience who said they had never repaired holes were asked why they did not repair the net and among those that replied 68% said they had no time, 20% said repairing was not necessary or holes too small, 6% said it was not possible, and 5% stated they had no materials to repair or did not know how to do it. Only one net in Pemba was reported to have been modified to enforce the border of the net.

### Survival in serviceable condition

**Fig. 4:**
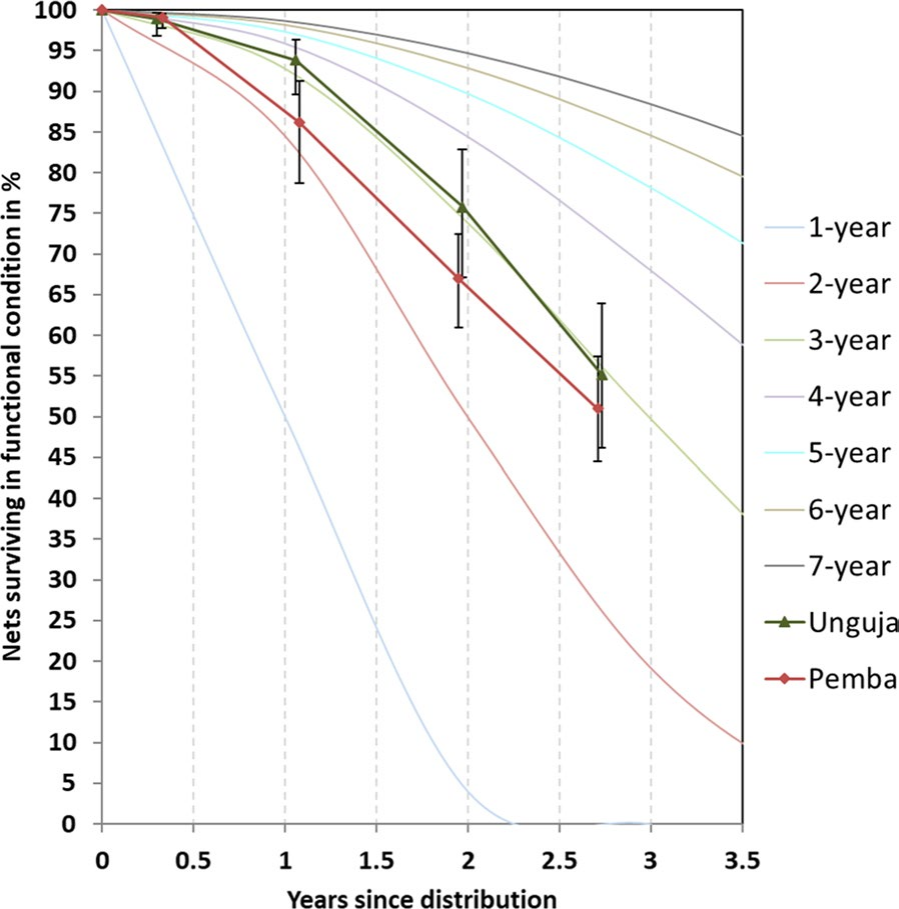
Survival of cohort nets in serviceable condition plotted against reference curves with defined median survival

**Fig. 5:**
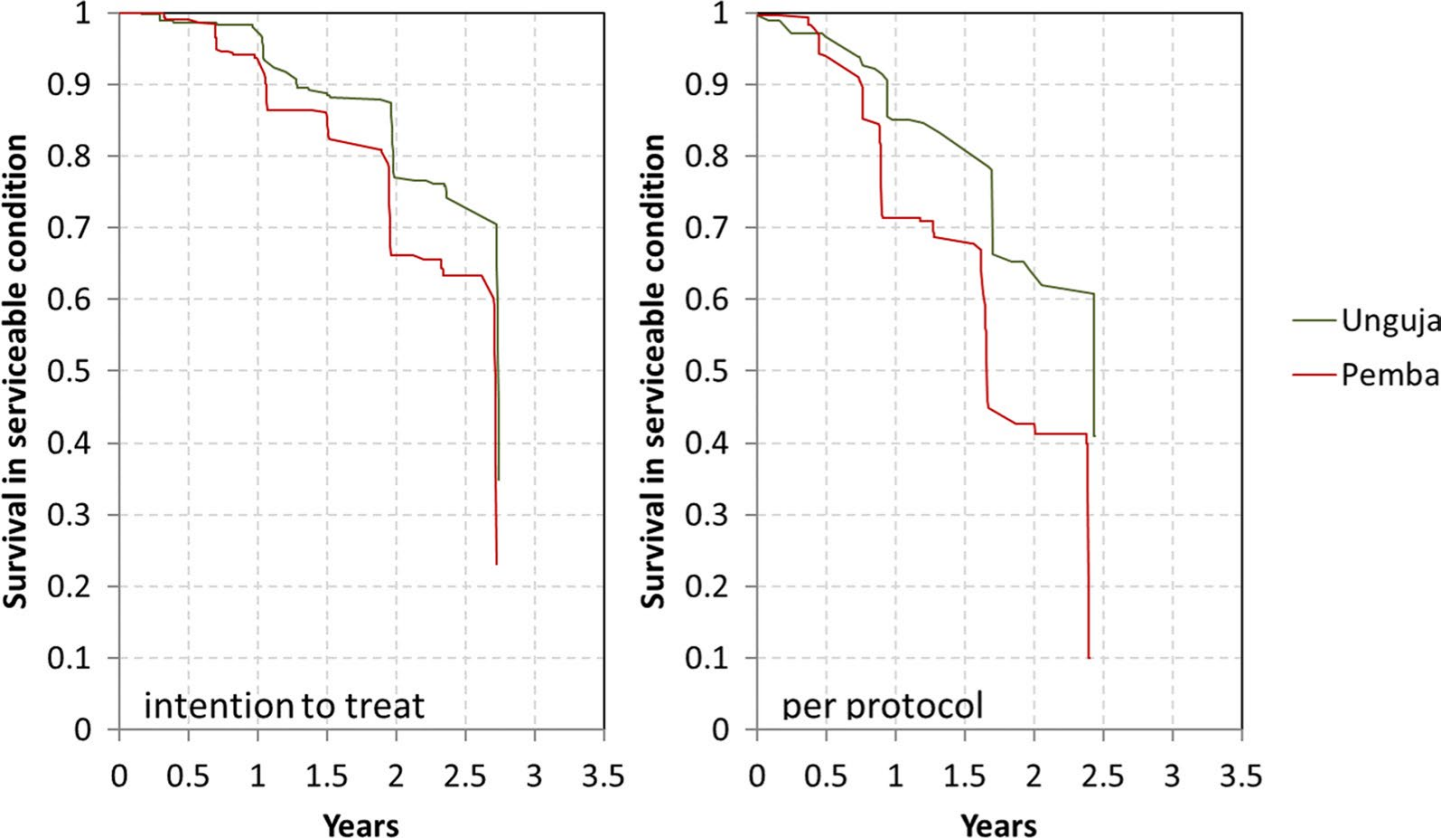
Kaplan–Meier survival functions of cohort nets comparing risk starting at distribution (intention to treat) versus starting at first hanging (per protocol)

The physical survival of LLINs in serviceable condition, i.e., combining attrition due to wear and tear and the integrity of the still existing LLINs, decreased rapidly over time (Table 5). At 12 months follow-up there was an 8%-point lower survival rate of the Olyset^®^ nets in Pemba compared to the PermaNet^®^ 2.0 in Unguja and a 9%-point difference at 24 months. However, this difference decreased to just 4% points at 33 months bringing the two curves closer together (Fig. 4). While at none of the time points the survival differed statistically between sites, survival in Pemba was found to be lower when the entire data were considered in a Kaplan–Meier survival function using an intention to treat approach (p < 0.0001) (Fig. 5, left). When a per-protocol approach was used, i.e., risk of failure to survive in serviceable condition only started with the first hanging of the cohort net, the lower survival of campaign nets in Pemba was even more pronounced (Fig. 5, right). Estimated median survival in serviceable condition using the graphical method (see “Methods”) was 2.9 years at the final data point for Unguja (PermaNet^®^ 2.0) declining from 3.3 years at 12 months and 3.1 years at 24 months. Estimates for Pemba (Olyset^®^) showed the opposite trend with 2.9 years at the final survey compared to only 2.3 years at 12 months and 2.6 years at 24 months.

**Table 5:** Estimated survival and median survival in serviceable physical condition

Variable	12 months	24 months	36 months
Unguja			
% surviving in serviceable condition (95% CI)	93.9 (89.6–96.4)	75.8 (67.1–82.2)	55.2 (46.2–63.9)
Median survival in years			
Estimated from Fig. 4	3.3	3.1	2.9
Calculated from last two data points (95% CI)	–	–	2.9 (2.5–3.3)
Pemba			
% surviving in serviceable condition (95% CI)	86.1 (78.7–91.2)	67.0 (60.6–72.6)	51.0 (44.5–57.4)
Median survival in years			
Estimated from Fig. 4	2.3	2.6	2.7
Calculated from last two data points (95% CI)	–	–	2.7 (2.5–3.0)

The Cox proportionate hazard models looking at determinants of physical survival are presented in Table 6. In the multivariable model only considering household level factors there was strong evidence of a 2.3 times higher risk of failure to survive over time in Pemba (Olyset^®^) compared to Unguja (PermaNet^®^) controlling for other factors. In addition, having had a very positive net-care attitude score at least twice (but not only once) in the surveys was shown to have a significant protective effect with an adjusted Hazard Ratio (aHR) of 0.71. However, there was no correlation of the net care attitude with the exposure to net related SBC messages (p = 0.40) and adding message exposure did not improve the explanatory power of the model. A protective effect was also seen in households headed by persons with any secondary education (aHR 0.65). In contrast, the risk of failure to survive increased with the number of children under 10 years old in the household. No impact on survival was seen from socio-economic tertile, discussing net care within the household, or female-headed households.

**Table 6:** Determinants of physical durability (risk of failure to survive in serviceable condition) from Cox proportional hazard models

Variable	Adjusted hazard ratio (HR)	95% CI	p-value
At household level; N = 2722 obs/890 nets			
Site/Brand of LLIN			
Unguja (PermaNet 2.0)	1.00		
Pemba (Olyset)	2.34	1.78–3.08	< 0.0001
Net care attitude of household across surveys			
Had a very positive score (> 1.0) never or only once	1.00		
Had very positive score (> 1.0) at least twice	0.71	0.53–0.97	0.031
Number of children under 10 years in household			
None	1.00		
1–2	2.00	1.10–3.65	0.023
3 or more	3.15	1.83–5.42	< 0.0001
Educational status of head of household			
Non-literate or primary	1.00		
Secondary or higher	0.65	0.51–0.82	< 0.0001
At net level (nets ever hung) N = 2244 obs/697 nets			
Site/Brand of LLIN			
Unguja (PermaNet 2.0)	1.00		
Pemba (Olyset)	2.54	1.85–3.48	< 0.0001
Net care attitude of household across surveys			
Had a very positive score (> 1.0) never or only once	1.00		
Had very positive score (> 1.0) at least twice	0.62	0.43–0.88	0.007
Number of children under 10 years in household			
None	1.00		
1–2	1.60	0.84–3.02	0.15
3 or more	2.12	1.19–3.81	0.011
Educational status of head of household			
Non-literate or primary	1.00		
Secondary or higher	0.71	0.54–0.92	0.011
Folding up of hanging nets during day			
Always or at least sometimes	1.00		
Never	1.77	1.29–2.43	< 0.001
Users of net			
Children alone or with adults	1.00		
Adults only	0.73	0.56–0.97	0.031
Type of sleeping place			
Finished bedframe, mattress or mat	1.00		
Unfinished bedframe	0.72	0.56–0.93	0.013

*Obs* observations

Adding net level variables, i.e., reducing the analysis to nets ever hung, slightly increased the effect of the positive net care attitude (aHR 0.62, p = 0.007) as well as the difference between sites (aHR 2.54, p < 0.0001). Educational status of head of household and children under 10 years of age in the household remained relevant but evidence of their impact was much weaker, suggesting that in part they were just a proxy for specific behaviours that directly affect survival. These were use of a net exclusively by adults (aHR 0.73) and consistent use over a finished bedframe (aHR 0.72), but these two factors did not show significant correlation between them as one might expect. A negative effect on survival was identified by never folding the net up when hanging (aHR 1.77). Net-related variables that did not have a significant impact on physical survival were storing food or cooking in the sleeping room, and drying nets over fences or bushes. Model diagnostics showed that the assumption of a proportionate hazard was not violated.

### Insecticidal effectiveness

The target of sampling 30 campaign nets at each site for bio-assay testing was achieved at all time points and at both sites (no samples were taken at baseline). The results of WHO cone and tunnel tests are presented in Table 7. For the PermaNet^®^ 2.0 60-min knockdown remained very high at all time points while 24-h mortality declined over time from a median of 93 to 72% at the final assessment. Even without the tunnel test optimal insecticidal performance was above or equal to 90% at all time points. For the permethrin-treated Olyset^®^ mortality rates of pyrethroid-susceptible vectors were significantly lower and declining, reaching only 44% at the final survey. However, when the tunnel test was applied to samples failing the cone test, all samples showed optimal performance after 33 months of follow-up. There was no evidence that the campaign nets sampled at 12 and 24 months from outside the study cohort differed in hanging, use and washing behaviour from the cohort nets.

**Table 7:** Results from bio-assays using WHO cone test

Variable	12 months	24 months	36 months
Unguja–PermaNet 2.0	N = 30	N = 30	N = 30
Knockdown 60 min			
Mean (95% CI)	89.0% (83.9–94.3)	97.8% (96.6–99.0)	96.9% (94.1–99.7)
Median (IQR)	92.0% (84.0–98.0)	100% (96.0–100)	100% (96.0–100)
Mortality 24 h			
Mean (95% CI)	92.1% (88.3–95.9)	86.4% (80.5–92.3)	71.5% (66.0–76.9)
Median (IQR)	93.0% (88.0–100)	89.0% (82.0–96.0)	72.0% (64.0–80.0)
Optimal effectiveness			
Estimate (95% CI)	90.0% (63.5–97.9)	96.7% (77.7–99.6)	90.0% (63.4–97.9)
Minimal effectiveness			
Estimate (95% CI)	100%	100%	100%
Optimal effectiveness			
(incl. tunnel) Estimate (95% CI)	–	100%	100%
Minimal effectiveness			
(incl. tunnel) Estimate (95% CI)	–	100%	100%

Pemba–Olyset	N = 30	N = 30	N = 30
Knockdown 60 min			
Mean (95% CI)	86.7% (82.9–90.4)	77.9% (72.1–83.7)	87.4% (83.2–91.6)
Median (IQR)	90.0% (80.0–96.0)	80.0% (66.0–90.0)	93.0% (78.0–98.0)
Mortality 24 h			
Mean (95% CI)	76.6% (69.4–83.8)	55.9% (49.2–62.5)	47.9% (39.1–56.6)
Median (IQR)	77.0% (68.0–88.0)	52.0% (46.0–70.0)	44.0% (30.0–68.0)
Optimal effectiveness			
Estimate (95% CI)	53.0% (32.8–72.8)	20.0% (9.8–36.6)	50.0% (30.5–69.5)
Minimal effectiveness			
Estimate (95% CI)	96.7% (77.7–99.6)	76.7% (52.3–90.8)	90.0% (71.3–97.0)
Optimal effectiveness			
(incl. tunnel) Estimate (95% CI)	96.7% (77.7–99.6)	80.0% (58.4–91.9)	100%
Minimal effectiveness			
(incl. tunnel) Estimate (95% CI)	100%	96.7% (77.7–99.6)	100%

## Discussion

Comparing the physical and insecticidal durability of two LLIN brands, the polyester-based, 100 denier PermaNet^®^ 2.0 treated with deltamethrin and the polyester-based, 150 denier Olyset^®^ treated with permethrin, over a 3-year period this study found the physical survival in serviceable condition to be lower for the Olyset^®^ site at all time points. The difference was 8–9%-points at 12 and 24 months of follow-up and reduced to 4% points in the final assessment 33 months after distribution with survival in serviceable condition of 55% for Unguja (PermaNet^®^ 2.0) and 51% for Pemba (Olyset^®^). Estimated median survival at 12, 24 and 33 months varied between 3.1 and 3.3 years for the PermaNet^®^ 2.0 site and between 2.3 and 2.7 years for the Olyset^®^ site. The unadjusted Kaplan–Meier survival function by site/brand showed strong evidence for a significant difference (p < 0.0001). The design of the study was to undertake the comparison in similar environments in order to minimize the effects of other factors than the LLIN brand. The two selected districts on the islands of Unguja and Pemba of the semi-autonomous region Zanzibar were, indeed, very similar with respect to climatic, demographic and socio-economic characteristics. In addition, six out of the 12 household and net level potential risk factors for physical durability measured were also very similar and another four with some differences between sites (cooking in sleeping room, exposure to SBC messages, wash frequency and drying nets outside) were shown in the Cox proportionate hazard models to have no influence on the physical survival in this setting. This leaves two factors other than the brand that could have influenced the physical durability outcomes between the two sites. Folding up hanging nets during the day to take them out of harm’s way was significantly more common in Pemba, particularly during the last 2 years of the study. In contrast, a positive net-care attitude was more common in Unguja. Both these factors were significant determinants in the final Cox model of survival in serviceable condition but seem to have cancelled each other out as the crude Hazard Ratio of 2.50 for Pemba/Olyset^®^ compared to Unguja/PermaNet^®^ 2.0 only marginally changed in the final adjusted model to 2.54. This allows the inference that, adjusting for the existing differences between sites, survival of Olyset^®^ was significantly lower than that of PermaNet^®^ 2.0 in this setting with an approximately 6 months lower median survival.

The same two LLIN brands have previously been compared in a large durability study using the same methodology in mainland Tanzania undertaken in eight districts between 2013 and 2016 [7]. Estimated median survival in serviceable condition after 3 years was 2.0 years for Olyset^®^ and 2.5 years for PermaNet^®^ 2.0, i.e., a similar difference between the brands as in this study, but overall lower survival which was clearly below the assumed 3-year median survival. In contrast, a study in Zambia that also applied the most recent methodology found no difference between Olyset^®^ and PermaNet^®^ 2.0 distributed in 2011 and followed for 3 years and estimated the median survival for both to be 2.5 years [17]. An even lower estimated median physical survival of Olyset^®^ LLIN of 1.5–2.0 years was reported from Benin, but in this case no comparison with other brands was made [18]. These Olyset^®^ LLIN were also distributed in 2011. Another study from Siaya County, western Kenya sampled households that had received Olyset^®^ through a mass distribution 5 years earlier (in 2005) and assessed attrition as well as physical condition [19]. Although the authors did not calculate the survival in serviceable condition, the data they present allow an estimation suggesting that after 5 years 37% of the originally distributed nets with known outcome were still in serviceable condition at this site. This corresponds to median physical survival of approximately 4.0–4.3 years and demonstrates that survival of the Olyset^®^ LLIN brand beyond the 3-year mark is possible in some settings.

A direct comparison of durability field performance between Olyset^®^ and PermaNet^®^ 2.0 is also available from two other studies which, however, did not capture all elements of physical durability assessments and hence do not provide survival estimates. In Nampula Province, Mozambique, Olyset^®^ nets distributed in 2008 and still present in the sampled households were found to have more and larger holes than PermaNet^®^ 2.0 after one, 2 and 3 years of follow-up suggesting a poorer performance [20]. The opposite was found in camps of internally displaced people in Chad where 8% of surviving Olyset^®^ LLINs 1–2 years after distribution in 2007–2008 were no longer serviceable compared to 39% of PermaNet^®^ 2.0 or Interceptor^®^ LLIN [21]. However, in this case the polyester LLIN had 75-denier yarn and not 100-denier as in this study or the other studies cited above.

Based on the Mozambique study comparing Olyset^®^ and PermaNet^®^ 2.0 [20] as well as unpublished data from similar surveys in other countries, supported by the US President’s Malaria Initiative, that suggested that holes in the Olyset^®^ LLIN rapidly became larger once the first yarn break occurred (reported in [22]) the manufacturer of Olyset^®^, Sumitomo Chemicals, changed the knitting pattern to resolve this problem in 2013 [22]. This implies that this study and the Tanzania mainland study [9] which both found a significantly lower performance of Olyset^®^ compared to PermaNet^®^ 2.0 were done with the new knitting pattern Olyset^®^ LLIN while the study in Zambia [17] finding no difference between the two brands used the old knitting pattern thought to be more vulnerable. This suggests that hole formation and enlargement is a more complex process that depends on the interaction of a number of factors [23, 24] and more research is needed to fully understand these mechanisms and allow manufacturers to develop more durable netting materials.

Insecticidal durability in this study for the PermaNet^®^ 2.0 LLIN was excellent with mean 60-min knockdown rates over 95% in the WHO cone bio-assay tests at all time points and slightly declining mean 24-h mortality rates from 93% at 12 months to 72% at 33 months. This resulted in over 80% optimal insecticidal effectiveness after 33 months in accordance with WHO criteria [15]. For Olyset^®^ the cone bio-assay tests showed more steeply decreasing knockdown and mortality rates and by cone bio-assay alone 50% of the tested nets had optimal insecticidal effectiveness at the final survey. This is thought to be an effect of the higher repellency of permethrin compared to deltamethrin [25] which results in mosquitoes avoiding contact with the netting under the cone and landing on the plastic cone instead. This handicap can be overcome by either using a wire ball where mosquitos have no other choice than to contact the netting material [26] or the WHO recommended tunnel test [15]. Applying the latter, all Olyset^®^ samples had optimal insecticidal effectiveness after 33 months of follow-up. This demonstrates that insecticidal durability was also according to expectations for the Olyset^®^ LLIN. However, this only means that insecticidal protection was provided for pyrethroid-susceptible vectors and it has previously been shown that already in 2013 there was a significant level of pyrethroid resistance for the dominant vector *Anopheles arabiensis* at least on Pemba Island [3]. Therefore, the Zanzibar Malaria Elimination Programme is currently considering a shift to next generation LLINs, i.e., either LLIN with the addition of the synergist piperonyl butoxide (PBO) or a second active ingredient.

## Limitations

Some of the durability risk factors such as net care and repair attitude as well as some of the outcomes, such as reason for net losses, were based on the answers of the household members interviewed and, therefore, are prone to recall or social desirability biases. With the prospective design, there is also the potential for the Hawthorne effect, whereby being asked about net care and handling four times over the course of 3 years may have contributed to changes in behaviour. The standard durability monitoring approach tries to minimize this by conducting only four surveys vs every 6 months as had been done in some of the earlier studies. Furthermore, while the sample of the campaign net cohort was representative for the selected district within each island, the district selection was purposive and some caution is required when generalizing the findings to the Zanzibar as a whole. Finally, although during recruitment it was attempted to ensure that identified cohort nets were indeed from the most recent campaign, it cannot be completely ruled out that some were not.

## Conclusions

After 3 years of follow-up among similar, rural populations on the Zanzibar islands of Unguja and Pemba the 150-denier polyethylene LLIN Olyset^®^ showed significantly lower physical survival compared to the 100-denier polyester LLIN PermaNet^®^ 2.0 even after adjusting for other variables of net-use environment and net handling. This suggests that the differences were driven by the textile characteristics of the LLIN brands.

## Supplementary information

**Additional file 1.** Household characteristics. Contains table with demographic and socio-economic characteristics of sampled households.

## Data Availability

The datasets used and/or analysed during the current study are available from the corresponding author on reasonable request.

## Abbreviations

ANC: Ante-natal care
HR: Hazard ratio
IPC: Inter-personal communication
ITN: Insecticide-treated net
LLIN: Long-lasting insecticidal net
NMCP: National Malaria Control Programme
pHI: Proportionate hole index
SBC: Social and behaviour change
ZAMEP: Zanzibar Malaria Elimination Programme
