# Supplementary material for: Monitoring the durability of the long-lasting insecticidal nets Olyset® and PermaNet® 2.0 in similar use environments in Zanzibar
Source: Malar J. 2020 May 24;19:187. doi: 10.1186/s12936-020-03258-w (PMC7247136; doi:10.1186/s12936-020-03258-w)
Supplement: Supplementary file 1 — Additional file 1. Household characteristics. Contains table with demographic and socio-economic characteristics of sampled households. [file 12936_2020_3258_MOESM1_ESM.pdf]

Additional file 1

Table: Household characteristics (based on households that were seen at baseline and endline surveys, N=243)

Variable	Unguja	Pemba	P-value
	% (95% CI)*	% (95% CI)*	for comparison between sites
**Demographic**			
Mean number of de-jure members	5.4 (5.1-5.8)	6.1 (5.5-6.7)	0.053
Mean age of head of household in years	47.7 (45.0-50.4)	47.6 (43.8-51.3)	0.96
Proportion of female headed households	24.3 (17.8-32.4)	11.4 (6.5-19.4)	0.016
Proportion of under-fives	14.8 (12.8-17.1)	17.7 (14.8-20.9)	0.13
Education of male heads of household			
Non-literate	26.1 (19.0-34.5)	28.2 (19.6-38.8)	
Primary	33.2 (24.3-43.5)	27.0 (20.7-34-4)	0.60
Secondary	40.8 (31.6-50.9)	44.8 (33.9-56.2)	
**House characteristics**			
Improved roof materials	0.4 (0.1-3.1)	4.0 (0.5-26.7)	0.06
Improved wall materials	78.2 (62.1-88.6)	64.9 (46.9-79.6)	0.22
Improved floor materials	72.2 (58.9-82.4)	72.1 (56.4-83.8)	0.99
Cooking fuel kerosene or gas	0.8 (0.2- 3.2)	0.8 (0.2- 3.3)	0.96
**Water and sanitation**			
Access to safe water	84.5 (77.0-89.8)	89.9 (75.6-96.3)	0.39
Access to any latrine	93.4 (86.7-97.1)	82.7 (66.0-92.1)	0.06
Improved latrine or flush toilet	30.7 (18.1-47.09	47.2 (31.1-63.9)	0.15
**Household assets**			
Any transport	61.8 (52.7-70.0)	66.9 (55.6-76.6)	0.45
Type of transport			
Bicycle	57.1 (47.4-66.3)	60.9 (49.8-71.0)	0.60
Motorcycle	10.1 (6.0-16.6)	14.1 (9.6-20.4)	0.29
Car	3.4 (1.6- 7.1)	4.4 (2.0- 9.4)	0.60
Owns farm land	64.3 (49.5-76.8)	60.9 (49.6-71.2)	0.70
Owns livestock	64.7 (54.1-74.1)	71.8 (61.6-80.1)	0.30
Type of livestock			
Chicken	58.6 (46.5-69.7)	61.3 (49,5-71.8)	0.74
Duck or turkey	9.3 (6.5-13.2)	8.5 (4.4-15.8)	0.81
Goats	6.8 (3.2-13.7)	3.3 (1.1- 9.5)	0.26
Cows	11.0 (5.8-19.9)	13.6 (7.2-24.3)	0.62
Household items owned			
Radio	64.7 (59.6-69.5)	44.4 (36.6-52.4)	0.0001
Television	18.9 (11.8-28.9)	35.5 (23.2-50.0)	0.038
Refrigerator	14.7 (8.4-24.4)	26.2 (16.9-38.3)	0.085
Fan	12.6 (6.7-22.4)	22.2 (13.3-34.7)	0.14
Iron	13.5 (6.9-24.7)	27.4 (16.8-41.4)	0.067
Any mobile phone	89.1 (82.3-93.5)	84.7 (75.8-90.7)	0.32
Smartphone or computer	13.5 (9.3-20.2)	24.2 (15.9-35.0)	0.050

*Unless otherwise indicated in column 1
